# Hospital and Institutional News

**Published:** 1913-05-03

**Authors:** 


### HOSPITAL AND INSTITUTIONAL NEWS.

### INCREASED PAY FOR MENTAL WORKERS.

We publish elsewhere in this issue the chief features of the new scheme under which the staffs of the Metropolitan Asylums Board receive increased salaries. The new proposals will come into force on June 1, and affect not merely the salaries, but the hours of the staffs. Those who are in favour of the living-out system and its extension will note that all laundry-maids are to have the option of living out and of receiving an allowance in lieu of board, lodging, and washing. It will be noticed that the new scheme applies to other branches of the Board's service, as far as that is possible, where officers of the same grades are employed. The new scheme will be welcomed on the two grounds that it has resulted from the presentation of a petition by the mental hospital staffs to the Board, and that it is a further step on the road towards making the salaries and emoluments of mental staffs more commensurate with the great strain of their work and its highly exacting duties.

### LIND-AF-HAGEBY v. SALEEBY AND THE "PALL MALL GAZETTE."

After an inordinately lengthy trial, chiefly due to the verbosity of the plaintiff, the jury found for the defendants in this case, and the Judge went out of his way to express his conviction that no other verdict would have been satisfactory. It might be thought that this case, together with Dr. Bayliss' successful action against Mr. Coleridge ten years ago and the Report of the Royal Commission in between, would suffice to convince anti-vivisectionists that informed opinion (lay, not medical) is so adverse to their claims, their propaganda, and their methods that some alteration thereof would be advisable. But anti-vivisectionists are a law unto themselves, and we note that already Mr. Coleridge has announced publicly that the result of the trial will make no difference to the work of his Society. To do him justice, Mr. Coleridge is a less unreasonable person than Miss Lind-af-Hageby, but all the fraternity are infected with the same inability to look plain facts in the face and understand them, and probably the old campaign of mendacity and calumny will go on just as merrily as before. Meanwhile we offer best congratulations to all the defendants. The *Pall Mall Gazette* has deserved well of the public by standing up for truth and progress, and has received the good wishes of practically every daily paper in the country, except, of course, the anti-vivisectionist *Daily News*.

### THE SANITARY REGENERATION OF DELHI.

One curious fact about the existing City of Delhi, which is of interest now that here has been fixed the site of the new Indian capital, is that the death-rate is higher than the birth-rate. At the time when the site of the capital was undecided on the insalubrity of the present city was much discussed, and it is interesting to learn from the *Times* that a health officer is already at work. The Imperial Government and the municipality of the city have appointed Major Cook Young, of the Indian Medical Service, to this post, and a sanitary committee has been formed. Major Young has been studying the health department of the Bombay Municipality, which will probably form the model on which the permanent sanitary administration of the city will be set up. A sum of £33,000, or five lakhs of rupees, was included in the recent Indian Budget for starting the work. How far the relatively high death-rate is due to the alleged insalubrity of the city is unapparent, but the additional fact that 37 per cent. of infants died in the last statistical year before attaining the first year of life makes it appear that other causes, besides want of sanitation, have contributed to this result.

### NEW TUBERCULOSIS SCHEME FOR MIDDLESEX.

For the treatment of tuberculosis in Middlesex the Public Health Committee of the County Council will shortly launch a complete scheme for the division of the county into five separate areas with a main dispensary and sub-centres in each. Tuberculosis medical officers having special experience in phthisis are to be appointed for each dispensary area, together with the necessary staff of nurses and other officers. One of the first of the five medical officers to be selected is Dr. E. E. Norton, for a number of years medical superintendent at the Brentford Union Infirmary at Isleworth, who a few days since tendered his resignation to the Brentford Guardians in consequence. It is interesting to add that Dr. Norton, who begins this week a very instructive series of articles on "Milk and Milk-Contracts" in our columns, will probably be working as tuberculosis officer in the district in which the Isleworth Infirmary is situated.

### INSURANCE ACT AND GAINSBOROUGH DISPENSARY.

After a heated discussion it has been decided to give power to increase the subscriptions of the members of Gainsborough Dispensary. In consequence of the Insurance Act 1,600 members, it is stated, have been taken away, and thus the institution cannot be kept open unless the remaining members agree to increase their subscriptions. Two meetings broke up after refusing to give power to increase the subscriptions, and it was only on calling a third, after much excitement, that it was agreed that this should be done. The problem, however, is the wider one of the place of dispensaries generally under the National Insurance Act. If the members believe that the panel system provides, as it professes, adequate treatment for all diseases of the out-patient class, then the dispensaries' work has been replaced. The fact that the members of dispensaries all over the country do not feel that the dispensaries' work is being done by the Act, and are even willing to raise their subscriptions to preserve their institutions, makes the "contributions" appear as the tax that they are, and the "benefits" as the *quid pro quo* which they are not.

### INSURANCE DIFFICULTIES AT CARDIFF.

The question of policy as regards the Insurance Act is producing much bitterness between two sections of the medical men at Cardiff. The first nineteen medical men who bowed to the yoke of panel servitude were practically boycotted by the rest of the profession. The present dispute concerns the direct representation of the profession on the Insurance Sub-Committees for the City. Dr. James Robinson, chairman of the Cardiff Health Committee, and Dr. R. J. Smith support the application of the local branch of the British Medical Association that its two nominated representatives should be appointed to the Sub-Committee, on the ground that this direct representation is due to the profession under the Act. The Insurance Committee, on the other hand, argue that the present Committee is provisional and can be re-formed at the end of the first year of the Act. In addition to this source of friction the South Wales and Monmouthshire Branch of the British Medical Association has passed a resolution accusing the Insurance authorities of a breach of faith in respect of the medical-aid associations which have been formed.

### MARGARINE AT MENTAL HOSPITALS.

The decision of the West Riding Asylums Board to economise by substituting margarine for butter in the food supplies to staffs and patients at the Board's four institutions has received general condemnation throughout the West Riding of Yorkshire, on the part not only of the organised and unorganised mental hospital attendants and nurses, but of many Boards of Guardians who send patients to these institutions, who have given hearty support to a resolution of protest which has been circulated by the Wakefield Trades and Labour Council. The Guardians consider that as they have to pay for the maintenance of large numbers of Union patients in the mental hospitals they have a right to expect the patients to be given the best food. The Asylums Board's action has been freely spoken of as a mean and paltry method of saving. The staff at Storthes Hall Asylum, who were the first subjects of the change of policy, immediately "struck," and since April 10 they have eaten bread without any "butter." The patients, of course, have had no choice in the matter. On Saturday last a meeting of the Yorkshire Centre of the National Asylum Workers' Union was held at Leeds, and a statement was afterwards made to the effect that two Labour members of the Asylums Board had attended and informed the meeting that the Chairman of the Board had assured them that in future no margarine would be issued to any of the staffs. The meeting passed a resolution asking the Board to rescind its decision and to grant the attendants and nurses of Storthes Hall money in lieu of the butter during the time the "strike" had been in progress. It was decided that the Union's solicitors should take action if the Board's reply proved unsatisfactory.

### SANATORIA AND THE LONDON COUNTY COUNCIL.

The Chairman of the Finance Committee of the London County Council, Mr. R. C. Norman, in his annual budget statement at last week's meeting, made some interesting remarks as to the position of that body in the future treatment of tuberculosis. He pointed out that there is provision in the estimates for expenditure on the provision of sanatoria under the National Insurance Act. An expenditure of £45,500 is estimated for under this head, but a receipt of the same amount from the London Insurance Committee is included in the income. Arrangements have been made by the Council, with the co-operation of the Metropolitan Asylums Board, for the reception of insured patients in institutions set apart for that purpose by the Board, but as funds are provided under the Insurance Act for such persons they should occasion no charge on the county rates. Mr. Norman emphasised, and the ratepayer will do well to bear in mind, the fact that the Council has undertaken no financial responsibility in this matter. So far as the rest of the community is concerned, as mentioned in The Hospital last week, a scheme is now being prepared by the Public Health Committee of the Council, at the invitation of the Local Government Board, for dealing with tuberculosis throughout the county, and this will no doubt co-ordinate the work which is being done under the Insurance Act with all other efforts for contending with the disease. Mr. Norman, however, did not hold out much hope that the scheme will mature very quickly, but he pointed out that the County Council and the State are at present joint partners in the work of administration in many departments where both national and local interests are involved. The State, moreover, occupying for the most part the privileged position of a partner with limited liability.

### THE SUCCESS OF THE RADIUM APPEALS.

It is not only the London hospitals which are benefiting from the appeals for radium which have been issued of late. In addition to the gifts received for this purpose at Charing Cross Hospital and at the Middlesex, the Royal Infirmary, Liverpool, has received the £2,000 asked for to purchase radium for its *x*-ray department. This is believed to be the largest sum raised for the purchase of radium in any provincial town, and, whether it be so or not, Liverpool has set an example and shown a way that may well be followed in every provincial centre.

### THE MORTUARIES OF CORONERS' COURTS.

The report recently issued by the Coroner for the City of London and the Borough of Southwark, Dr. F. Joseph Waldo, is an extremely instructive document and full of interesting and practical suggestions for safeguarding life and for the improvement of the conduct of the investigations for which he is responsible. The suggestions carry all the more weight for the reason that they are based upon actual experience and the findings of the Coroner's juries. Amongst many important features dealt with in the report, such as the causes and prevention of fatalities due to the traffic of motor vehicles, the result of the personal administration of the City of London Fire Inquests Act, the further safeguarding of workers in buildings, docks, and other occupations coming within the purview of the Factory and Workshop Acts, the following important references are made by Dr. Waldo. He states that the Herscher's apparatus for the preservation of bodies, which was installed in the City Mortuary in 1909, and is the only apparatus of the kind associated with any Coroner's Court, has proved of great service in the identification of bodies. A similar apparatus is much needed for the Borough of Southwark in view of the large number of unidentified bodies of those found drowned in the Thames. Dr. Waldo points out, as he has repeatedly done in the past, the necessity for a further improvement in the Coroner's Court at Southwark, for, owing to the position of the mortuary—half a mile away from the Court—much valuable time is wasted at each inquiry. The worst feature is, however, he states, the insanitary and dangerous condition, structurally, of the mortuary. There is plenty of room for an up-to-date mortuary in the immediate precincts of the Court, whereas the proposed rebuilding of the Court and mortuary on the old burial-ground of the Church of St. George the Martyr would appear to involve the Borough in an unnecessary expense, and, further, much delay would ensue in obtaining the necessary Act of Parliament sanctioning the use of the burial-ground for building purposes.

### PANEL SERVITUDE IN LONDON.

Even the London Insurance Committee has been unable to shut its ears to the statistical evidence on which the most grave and least sensational charges of sweating panel doctors and of cheating the insured have been based. At a meeting last week a statement was presented showing the number of panel doctors with more than one thousand insured persons as patients. Mr. Rockliffe was ruled out of order in attempting to move that doctors with more than two thousand patients should be prevented from accepting any more, and thereupon very nearly succeeded in turning the tables upon Mr. J. A. Dawes, the chairman, by moving the adjournment of the Committee as a protest. In the course of his speech for the adjournment Mr. Rockliffe referred to the issued circular which stated that eighty doctors have over two thousand, and some over four, five, and even six thousand patients. This, he added, was a monstrous state of things from the point of view of the insured. Evidently many members of the Committee agreed with this, for his motion for adjournment was lost by a narrow majority only. The Medical Benefit Sub-Committee recommended that "medical practitioners be permitted to join the panel for the purpose of treating a limited number of insured persons," which was agreed to. This recommendation, by which it is hoped many medical men with a private practice too large to be thrown away, but not too large to prevent additional sources of income from being welcome, will join the panel who have hitherto held aloof, was allowed to pass without protest from the chair because it suits the Commissioners. Mr. Rockliffe's motion to limit the number of each panel practitioner's patients to two thousand was ruled out of order because it does not help the Committee, but only the unfortunate insured.

### HANDBOOKS FOR HOSPITAL SUNDAY.

In London this year Hospital Sunday falls upon May 25, and it is time again to consider in what way best to remind the congregations of London of their responsibilities to the voluntary system. Last year, of course, this fact was brought home in the most personal manner by the private visit of their Majesties to St. Paul's Cathedral, which set an example that nothing else could have done. The provincial cities have lately been trying new expedients to re-awaken the early ardour of their clergy and congregations on behalf of this fund. Hull, for example, is considering the question of circulating 20,000 copies of a handbook, thus acting on the principle which has inspired The Hospital for so many years to distribute a Hospital Sunday Special Number to the churches on each anniversary. If this handbook be carefully thought out and well written and illustrated it might be of great value, and indeed of permanent interest. We shall hope to receive a special copy for review, and Hull has an opportunity of giving a practical hint to other provincial cities of which we hope it will make the best use. Such handbooks, as Mr. G. F. Grant has pointed out, should contain information about the institutions and contrast the support that they receive with the new duties thrust upon them of late years.

### COTTAGE HOSPITALS AND WORKING MEN.

A recent instance of the generosity with which working men support hospitals is seen in the financial prosperity of Maryport Cottage Hospital, Cumberland. A little over a year ago the working men agreed to increase their subscriptions, and the past year's list of receipts shows an increase of £108 from this source. Such a result is generally due to the good relations which the secretary and the committee are able to establish between the hospital and the workmen, and Mr. F. Kelly, the honorary secretary, must share in the congratulations which the institution has lately received. Another source of satisfaction for which the administration deserve praise is the reduction in the expenditure, which has amounted to £63. Mr. F. Kelly has been re-elected honorary secretary and treasurer.

### NEW EXTENSION OF STOKE NEWINGTON INVALID HOME.

The Stoke Newington Invalid Asylum and Home Hospital for Women received a letter from Queen Alexandra at the opening of the Lister Ward and extension last week. Her Majesty is patron of the hospital, and reminded those present that Lord Lister was one of its founders as well as on the list of its consulting surgeons. The institution does important work as a home of rest for girls and women, especially of the servant class, who require rest, change, and nursing. The Chairman, Mr. J. P. R. Lyell, read Queen Alexandra's letter, and the Lord Mayor performed the opening ceremony and unveiled a tablet in commemoration of the event. He said that he hoped that they would be able to obtain some assistance from the City Companies, as the extension had been undertaken in order to keep pace with the latest demands of sanitary and hygienic science. The extension has cost some £1,500, of which one-third has been already subscribed. We trust that this extension of an old-established institution will give it a new lease of usefulness and attract the increased support which it deserves.

### OVERCROWDING AT CARMARTHEN MENTAL HOSPITAL.

A serious condition of overcrowding at the Joint Counties Mental Hospital, Carmarthen, is revealed in the report of the Lunacy Commissioners, Mr. A. H. Trevor and Mr. E. Marriott Cooke. They disclose a position in which there are 74 male and 46 female patients in excess of the proper accommodation. Dr. John Richards, the medical superintendent, accounts for the increase by a higher admission with a lower recovery rate. In the female epileptic dormitory the beds were much closer together than they ought to be, and eighteen patients were sleeping on the floor. At a meeting of the committee of visitors the medical superintendent's report was discussed, but the discussion mainly centred upon the disputes which have long arisen between Cardiganshire, Carmarthenshire, and Pembrokeshire, and the Home Office Award has not been carried out. A sub-committee has been appointed to arrange terms for a final settlement, but in the meantime the overcrowding appears to continue unchecked.

### A ROYAL PATIENT AND MEDICAL CONTROVERSY.

The death of Professor Fritz von Bramann, director of Hallé University Surgical Laboratory, removes the famous operator who attended the Emperor Frederick III. when Crown Prince, and whose operation on his august patient in February 1888 led to such embittered medical controversy. The English physician in attendance on the Prince was Sir Morell Mackenzie, who replied to the medical account of the illness which his German colleagues issued after the patient's death in a popular pamphlet. The performance of tracheotomy becoming inevitable Professor von Bergmann was appointed to undertake it, but in case of sudden suffocation his assistant, Dr. Bramann, remained at hand. Eventually called on at short notice to open the trachea, he insisted on waiting, since he had been kept from seeing the patient for several days previously, and eventually performed the operation successfully, although Sir Morell Mackenzie criticised as unskilful his insertion of the tube. The patient was suffering from cancer of the throat, and the German view has always been that the operation secured immediate if temporary relief.

### DOCTOR CHARGED WITH ILL-TREATING PATIENTS.

The serious charges of ill-treating mental patients and receiving them for payment in an unlicensed house contained in the summonses issued by the Public Prosecutor on behalf of the Commissioners in Lunacy against Dr. Henry Thomas Hamilton, of Barnes, have led to his being committed for trial at the Central Criminal Court. A large amount of evidence has been heard, but on the summonses respecting Miss Hay-Coghlan and Miss Hickson the Bench decided to commit. The defendant was admitted to bail.

### THE METROPOLITAN ASYLUMS BOARD AND THE INSURANCE ACT.

If the method adopted by the Metropolitan Asylums Board for carrying out their portions of the exactions of the Insurance Act proves a success, doubtless many Poor-Law institutions will follow suit. They propose to arrange for their own medical staff to go on the panel for the attendance of their employees, and for their dispensers to join the panel of dispensers under the Act. As the Metropolitan Asylums Board have realised the importance of engaging qualified pharmacists as their dispensers this will be in order, but if, and when, the idea occurs to the various Boards of Guardians who are not so particular, it will be found that all their dispensers are not eligible to sign the contract, not being pharmacists. The Public Pharmacists' Association are probably responsible for the origination of the proposal, for we must remember that some time ago they circularised the Guardians of the Metropolitan area asking that the pharmacists be allowed the privilege, doubtless for the purpose of increased remuneration.

### "THE CHILDER CHAINE."

We have often drawn attention before now to the value of a Ladies' Association, but it has been left to Mr. Thomas Gregg, secretary of the Belgrave Hospital for Children, to remind the public of the way in which a Ladies' Association can be of value in starting other fruitful organisations. In referring to a recent Note in The Hospital on "Encouraging Children to Support the Hospitals," Mr. Gregg draws attention to the organisation in connection with the Ladies' Association of his hospital, known as "The Childer Chaine." This is a Children's Association, formed in the early part of 1910 to help the Belgrave Hospital for Children, Clapham Road. It consists of ordinary members, who are children under sixteen years of age, and associate members, adults or children who, after attaining that age, desire to continue their association with it. The annual subscription consists of half-a-crown, and members are encouraged by the presentation of badges and badges of honour to increase the number of links by introducing new members, all of whom are elected by the committee. Competitions, sales, concerts, and other entertainments are held on behalf of the funds. The "Chaine" owes a very great deal to the personal service and enthusiasm of the Honorary Secretary, Miss May Carlos, and that of Mr. T. F.

Rider, a member of the hospital's committee, who has added to his previous services by bringing out a "Childer Chaine" Calendar, which was the means of adding ten guineas to the funds. In February the membership totalled three hundred.

### VICISSITUDES OF A CHILDREN'S HOSPITAL.

The Princess Royal paid a visit to the Belgrave Hospital for Children, Clapham Road, on Saturday afternoon to re-open the Babies' Ward. Since its removal to South London the hospital has undergone many vicissitudes. Lack of funds and the heavy payments yearly off the building account crippled the resources of the hospital, and three years ago the governors were compelled to close two wards and the out-patients' department. Happily, the latter was soon re-opened, and twelve months ago one of the wards was again available for patients, whilst this year the Princess Royal had the satisfaction of realising that she could once again throw open the whole of the institution to relieve the sick children of South London. The ladies have done splendid service for the hospital, Mrs. Bourchier Hawksley alone collecting no less than £1,000. A special appeal made by Mrs. Kendal realised £71 on Saturday, and, in addition, purses containing £125 were presented to the Princess by the children.

### NINE MONTHS OF SANATORIUM BENEFIT.

In a letter to the lay Press Dr. A. W. George draws attention to the way in which the provisions of the Insurance Act in respect of the treatment of tuberculosis are working after nine months of activity. He then quotes the case of an ex-Army man, employed as an indoor servant, who, on March 21 or 22, not being well, consulted his panel doctor, who diagnosed lung trouble. Another doctor was consulted, who communicated with the man's mistress, and eventually Dr. George was called in. Positive evidence of rather advanced pulmonary tuberculosis was forthcoming, and on March 28 application was made, marked "Urgent," to the London Insurance Committee for sanatorium treatment. A week later this was acknowledged, and on the Committee's request their expert was visited on April 7. After three days the man is told that "arrangements will be made" for his admission to an institution, which further promise of delay so much disgusted the man's friends that Dr. George "made application for his admission to the Hampstead Infirmary, where, of course, he could have gone at first." This is another example of how the voluntary hospitals make good the disastrous deficiencies of the Act.

### A CHEST HOSPITAL AND INSURED PATIENTS.

The effect of the Insurance Act upon the Royal National Hospital for Consumption has been swift and extensive. The number of waiting patients, as Mr. P. B. Burgoyne remarked at the annual meeting, has fallen from seventy or eighty to ten, and last year's record in the matter of subscriptions has fallen off this year. It has, therefore, been decided to admit insured patients to the extent of the surplus capacity of the hospital. Governors' nominees are to have the prior claim and will pay ten shillings a week as hitherto. An insured person admitted on a governor's letter of recommendation will be charged 25s. a week, and one admitted without a governor's letter, but merely on the recommendation of an Insurance Committee, will be charged £2 a week. It will be interesting to see whether this scheme works successfully and how far it is possible to have a special class of State-supported patients in the ordinary wards of the voluntary hospital.

### NEW SUPERINTENDENT AT SALOP MENTAL HOSPITAL.

Dr. William Stanley Hughes, medical superintendent of North Wales Counties Mental Hospital, Denbigh, has been appointed medical superintendent of the Salop Mental Hospital, Becton, Shrewsbury. Mr. Hughes took the conjoint diploma in 1903, having studied at Cardiff and St. Mary's Hospital. Before going to the Denbigh institution he held the post of assistant medical officer at Claybury Mental Hospital, Woodford Bridge. He is, of course, a member of the Medical Psychological Association.

### EARLSWOOD MENTAL HOSPITAL AND THE FEEBLE-MINDED.

Sir Frederick Mirrilees, who presided at the annual dinner of subscribers to the Earlswood Mental Hospital last week, referred to the grants proposed in the Government measure for the care of the feeble-minded. The suggested grants, he said, would be sufficient only for 10 per cent. of the persons estimated by the Royal Commission to be in need of care and control. He added that some £56,000 had been spent on structural improvements at the hospital, but more money was required for better appliances. If the proposed measure be carried in anything like its suggested form we believe that the numbers of persons affected by it will necessitate a further classification of patients in existing institutions and of the various institutions themselves. For those designed for serious cases in many respects are not suitable places for the loosely defined diathesis which feeble-mindedness yet appears to be.

### PUBLIC DISPENSER COMPLETES THIRTY-SIX YEARS' SERVICE.

Mr. R. E. Jones has resigned his post as pharmacist to the Poplar Board of Guardians, after serving for the long period of thirty-six years. The Guardians have added four years to the service as a token of the esteem Mr. Jones has merited, and his colleagues have presented him with a silver-mounted umbrella, a cigarette case, and a purse of gold. Having taken an active part in connection with the Public Pharmacists' Association, he was elected an honorary member. Mr. Jones qualified as a chemist and druggist in 1876, and was also a registered dental surgeon. We notice that the Local Government Board do not intend to permit the increased salary to public dispensers without the letter of their Standing Order being fulfilled. The Southwark Board of Guardians, recommending that a £10 increase should be granted to Mr. J. F. Dunstan, have been reminded by the Board that only two and a half years have elapsed since the £180 maximum was reached, and they ask for information as to the exceptional circumstances which should justify the increment.

### OSPITAL PHARMACIST BECOMES BARRISTER.

Recent years seem to indicate a tendency for pharmacists to acquire forensic distinction, there being nearly a dozen now on the roll. One of the latest, whom we heartily congratulate, is Mr. Reginald R. Bennett, pharmaceutist at the University College Hospital. Mr. Bennett was a scholar at the Pharmaceutical Society's School, and took no fewer than seven medals whilst there. He graduated B.Sc. and became a Fellow of the Institute of Chemistry, and this month was called to the Bar at Gray's Inn. He is an honorary secretary of the British Pharmaceutical Conference, which holds its Jubilee Meeting this year in London.

### THE BRITISH PHARMACEUTICAL CONFERENCE.

The third Conference of its kind to meet in London opens with a reception at the Guildhall on July 21. The first meeting was held at Newcastle in 1863, and since then most of the important centres have been the places of meeting. Both the Colonies and foreign countries will be represented, and the fifty years which have passed since the first meeting show that, in spite of carping critics, such conferences are not merely enjoyable and useful, but actively promote developments in the subject round which they centre.

### THE ALLEGED CREDULITY OF MEDICAL MEN.

The most noteworthy feature of the additional evidence given by Mr. G. J. Parry at a recent sitting of the Select Committee on Proprietary Medicines, was a number of small criticisms of the medical profession, the cumulative effect of which amounted, in the opinion of the Chairman, to a very remarkable charge. He said that the education of many practitioners regarding the use of drugs depended upon the firms which made the drugs, their travellers, and their literature. An enormous amount of money was, Mr. Parry asserted, spent upon travellers, whose sole duty was to call upon doctors to induce them to prescribe the compounds of particular firms; he thought medical men were very credulous with regard to these articles. To some extent this was due to the fact that the education of the doctor in pharmacy, chemistry, and materia medica was totally inadequate. It was anticipated that the medical practice of prescribing proprietary compounds would be used by the proprietors as a weapon of defence, but so vast is the difference between the class of products usually introduced to the prescriber by manufacturing chemists of high reputation and the average "patent medicine" advertised to the public that comparison is impossible.

### ENORMOUS CONTINGENT BEQUESTS.

The will of Colonel Frank Shuttleworth, an almost millionaire ironfounder and Great Northern Railway director, contains provisions which may conceivably some day bring to British hospitals an enormous accession of funds. To begin with, the Bedford County Hospital is to have £3,000; the Lincoln County Hospital £2,000; the Middlesex Hospital and the Cancer Hospital £1,000 apiece; and the Great Northern Hospital and the Dispensaries at Bedford and Lincoln £500 each. These bequests take effect immediately; but there are further and much larger contingent legacies. Should the testator's son not live to reach the age of twenty-three years, and should there be no other direct heir, one half of the residual estate goes to his cousins, and the other half—about £400,000—to such charitable objects or institutions as the executors in their sole discretion may decide. If this provision of the will should become operative the task of the executors will be a colossal one indeed. We note also that by the will of the late Mr. Edward Webb the Corbett Hospital, Amblecote, Staffordshire, receives £3,000 for a memorial wing to the testator's wife and £2,000 for the equipment and furnishing of the same.

### THE SALE OF DISINFECTANTS.

On Thursday, May 1, liquid disinfectants containing a small proportion of carbolic acid became included in the category of poisonous substances which when sold by retail must be labelled with the name and address of the seller and the words "Poisonous—Not to be Taken," and must be sold in bottles distinguishable by touch from ordinary bottles. Hitherto the sale of preparations containing less than 3 per cent. of carbolic acid has not been subject to any restriction, and, as a consequence, poisonous disinfectants have sometimes been given out to the public in beer bottles and other vessels. In hospital dispensaries precautions have always been observed in the storage of poisonous substances, and the new regulations will not affect the existing practice.

### THIS WEEK'S DRUG MARKET.

There has been a slight improvement in the demand for drugs, but business is far from active. Few important changes in prices have taken place. Oil of almonds is much dearer owing to the difficulty of obtaining almonds. A fair business has been done in opium, morphine, and cocaine, and there is a firmer tone in these three drugs; the demand results from the proposal to raise the import duty on them in the United States, and most of the buying has been for America. Cod-liver oil still tends lower in price, and the demand is very dull; there has been some improvement in the fishing at Finmarken, but the total yield at the end of the season, for the whole of Norway, cannot be much more than half of last season's yield. Essence of lemon is again tending dearer. Menthol is, if anything, inclined towards lower prices, and the same may be said of sugar of milk.

